# High drug resistance among Gram-negative bacteria in sputum samples from an intensive care unit in Nepal

**DOI:** 10.5588/pha.21.0034

**Published:** 2021-11-01

**Authors:** R. Ghimire, H. A. Gupte, S. Shrestha, P. Thekkur, S. Kharel, H. P. Kattel, P. S. Shrestha, N. Poudel, S. Shakya, S. Parajuli, A. Mudvari, J. Edwards

**Affiliations:** 1 Maharajgunj Medical Campus, Tribhuvan University Teaching Hospital, Kathmandu, Nepal; 2 Narotam Sekhsaria Foundation, Mumbai, India; 3 World Health Emergencies Programme, WHO Country Office, Kathmandu, Nepal; 4 Centre for Operational Research, International Union Against Tuberculosis and Lung Disease (The Union), Paris, France; 5 Centre for Operational Research, The Union South-East Asia Office, New Delhi, India; 6 International Friendship Children’s Hospital, Kathmandu, Nepal; 7 Department of Microbiology, Tribhuvan University Teaching Hospital, Kathmandu, Nepal; 8 Department of Anaesthesiology and Critical Care, Tribhuvan University Teaching Hospital, Kathmandu, Nepal; 9 Central Department of Public Health, Institute of Medicine, Tribhuvan University, Kathmandu, Nepal; 10 Department of Global Health, University of Washington, Seattle, WA, USA

**Keywords:** SORT IT, AMR, *Pseudomonas*, *Acinetobacter*, *Burkholderia*, *Stenotrophomonas*

## Abstract

**SETTING:**

Tribhuvan University Teaching Hospital, Kathmandu, Nepal.

**OBJECTIVES:**

1) To report the number and proportion of *Pseudomonas, Acinetobacter, Burkholderia*, *Stenotrophomonas* (PABS) species among intensive care unit (ICU) patients with sputum culture; and 2) to assess antimicrobial resistance patterns, demographic and clinical characteristics associated with resistance to at least one antibiotic and ICU discharge outcomes among those patients with PABS species admitted to hospital between 14 April 2018 and 13 April 2019.

**DESIGN:**

This was a hospital-based, cross-sectional study using secondary data.

**RESULTS:**

Of 166 who underwent sputum culture, 104 (63%) had bacterial growth, of which, 67 (64%) showed PABS species. Of the positive cultures, *Pseudomonas, Acinetobacter, Burkholderia* and *Stenotrophomonas* were present in respectively 32 (30.7%), 31 (29.8%), 1 (1%) and 3 (2.8%). *Pseudomonas* showed a high level of resistance to levofloxacin (61%), cefepime (50%) and amikacin (50%). *Acinetobacter* was largely resistant to cefepime (95%), imipenem (92%) and levofloxacin (86%). Of the 67 with PABS infection, 32 (48%) died.

**CONCLUSION:**

The study showed a high prevalence of *Pseudomonas* and *Acinetobacter* and the emergence of *Stenotrophomonas* in sputum culture samples of ICU patients. This highlights the need for monitoring PABS and associated resistance patterns to reduce mortality in ICU patients.

## Abstract (FR)

**LIEU:**

Hôpital universitaire de Tribhuvan, Katmandu, Népal.

**OBJECTIFS:**

1) Rapporter le nombre et le pourcentage d’espèces de *Pseudomonas, Acinetobacter, Burkholderia* et *Stenotrophomonas* (PABS) parmi les patients admis en soins intensifs (ICU) avec culture d’expectorations ; et 2) évaluer les profils de résistance antimicrobienne, les caractéristiques démographiques et cliniques associées à la résistance à au moins un antibiotique et le devenir des patients après un séjour en ICU parmi ceux infectés par une espèce PABS admis à l’hôpital entre le 14 avril 2018 et le 13 avril 2019.

**MÉTHODE:**

Étude transversale hospitalière réalisée en utilisant des données secondaires.

**RÉSULTATS:**

Sur 166 patients dont les échantillons d’expectorations ont été mis en culture, 104 (63%) présentaient une croissance bactérienne dont 67 (64%) étaient associées à la présence d’espèces PABS. Parmi les cultures positives, PABS étaient présents dans respectivement 32 (30,7%), 31 (29,8%), une (1%) et trois (2,8%) cultures. *Pseudomonas* a été associé à un niveau de résistance élevé à la lévofloxacine (61%), à la céfépime (50%) et à l’amikacine (50%). *Acinetobacter* était majoritairement résistant à la céfépime (95%), à l’imipénème (92%) et à la lévofloxacine (86%). Sur les 67 patients présentant une infection à PABS, 32 (48%) sont décédés.

**CONCLUSION:**

L’étude a montré une forte prévalence de *Pseudomonas* et *Acinetobacter,* ainsi que l’émergence de *Stenotrophomonas* dans les échantillons de culture d’expectorations des patients admis en ICU. Cela souligne le besoin de suivi des espèces PABS et des profils de résistance associés afin de réduire la mortalité des patients admis en ICU.

Antimicrobial resistance (AMR) is a global public health problem that is impacting environmental, social and economic targets of the UN Sustainable Development Goals (SDGs).^1^ Infections due to drug-resistant pathogens claim the lives of at least 700,000 people per year worldwide and this will likely rise to approximately 10 million by 2050.^2^ In response, the WHO has endorsed a Global Action Plan (GAP) on AMR for strengthening capacities of countries to tackle drug resistance.^3^

A risk assessment conducted by the WHO has demonstrated that AMR has been extremely challenging in the South-East Asian Region (SEAR).^4^ These challenges are particularly difficult for low- and middle-income countries (LMICs), where AMR is more prevalent, especially within tertiary intensive care settings. AMR now contributes to longer hospital stays, increased costs, morbidity and mortality.^5^ Multiple broad-spectrum antibiotics are routinely used empirically to manage life-threatening infections. The irrational use of antibiotics has contributed to the greater emergence of drug-resistant pathogens in developing countries.^6^ Nepal is not an exception, where there has been a rise in AMR in recent years.^7^,^8^

Critically ill patients admitted to intensive care units (ICUs) are highly vulnerable to infection, as they often undergo numerous, repetitive invasive procedures.^9^,^10^ The ICU is often recognised as the nidus for infectious diseases within a hospital setting.^11^ Lower respiratory tract infections (LRTI) are the most common bacterial infections that occur in 10–25% patients admitted to ICUs, resulting in high overall mortality that ranges from 22% to 71%.^12^,^13^ Nearly 15% of all Gram-negative bacilli isolated from clinical specimens, including sputum, are non-fermenting Gram-negative bacillus (NFGNB).^14^ Predisposing factors such as prolonged antimicrobial therapy, older age, advanced surgical and medical treatment, can lead to infections by NFGNB.^15^

NFGNB in tertiary care hospitals are frequently multidrug-resistant (MDR).^16^,^17^ Among the NFGNB bacteria, the most frequently observed pathogens reported have been the *Pseudomonas*, *Acinetobacter*, *Burkholderia* and *Stenotrophomonas* (PABS) species.^16^,^18^,^19^ Their prevalence and patterns of resistance in developing countries have not been well-documented. As reported in several studies previously, the burden of lower respiratory tract infections (LRTIs) caused by PABS organisms is thought to be high in ICU settings of developing countries such as Nepal.^16^,^18^,^20^ In these studies, PABS isolation was more frequent in sputum specimens than other sources. To our knowledge, only a single study has been conducted on the prevalence and resistance pattern of *Stenotrophomonas maltophilia* in ICU settings in Nepal,^18^ despite increasing reports of *S. maltophilia* outbreaks within ICUs in other countries.^21^

Therefore, we conducted this study to determine the burden of antimicrobial resistance of PABS species among patients admitted in the adult ICU and undergoing sputum culture at a tertiary care hospital in Kathmandu over a period of 1 year. Specific objectives were to determine the number and proportion of patients with growth of PABS species in sputum cultures, and to assess the pattern of antimicrobial resistance, the demographic and clinical characteristic associated with resistance to at least one antibiotic and ICU discharge outcomes among patients with PABS confirmed on sputum culture.

## METHODS

### Study design

This was a hospital-based, cross-sectional study of routinely collected hospital data.

### Setting

The health system in Nepal (population: 30.2 million;^22^ 7 provinces and 77 districts) is characterised by a wide network of health facilities and community workers and volunteers. Only 62% of the Nepalese households have access to health facilities within 30 min, with a significant urban (86%) and rural (59%) discrepancy.^23^ The healthcare delivery institutions in the country include private, public and mixed institutions.

Tribhuvan University Teaching Hospital (TUTH), the first teaching hospital of Nepal (established in 1983), is a 700-bed public facility that accepts all patients regardless of payer source. Services provided include outpatient, general medical, surgical, maternal-child health, emergency, ICU and a broad array of supporting medical/surgical subspecialties.

The ICU at TUTH has 11 beds and is a mixed, medical-surgical unit with a high occupancy rate. General medical patients are the primary patients within the ICU setting, while neurosurgical patients are the most frequent surgical patients. There are approximately 800 patients admitted to the TUTH ICU every year.^24^ The ICU is staffed by full-time, in-house intensivists who provide full-spectrum intensive care, including invasive procedures and associated management. During the study period, the nurse-to-patient ratio was 2:1. There was a designated team for infection prevention and control within the ICU, which consisted of both a physician and nursing staff. Chlorhexi-dine-based hand sanitizers are at each bedside and staff are instructed to use these before/after each patient contact.

### TUTH laboratory

The TUTH laboratory has a full-time microbiology department, which receives all samples from both outpatient and inpatient departments, including the ICU. The specimens are transported to the microbiology laboratory directly after they are collected by ICU staff. Sputum specimens are inoculated onto 5% sheep blood agar, MacConkey agar and chocolate agar (HiMedia, Mumbai, India) and incubated at 35°C for 24–48 h.

## Study methods

### Identification of isolated organism

All isolates were identified based on their morphological appearance, Gram’s stain characteristics, catalase test, coagulase test, oxidase test and biochemical parameters per Clinical and Laboratory Standards Institute (CLSI; Wayne, PA, USA) guidelines.^25^ Only PABS bacterial isolates were included in the study; those isolates that had mixed bacterial species (PABS and others) were excluded.

### Antibiotic susceptibility testing

Antibiotics discs with specified concentrations are used to determine the susceptibility pattern of isolated organism by using the disk diffusion method (modified Kirby-Bauer method) on Mueller Hinton agar (Hi-Media) and interpreted according to CLSI guidelines. *Staphylococcus aureus* American Type Culture Collection (ATCC) 25923, *Escherichia coli* ATCC 25922 and *Pseudomonas aeruginosa* ATCC 27853 were tested in every set of the experiment in parallel as part of quality control.

### Antibiotic susceptibility

Antibiotic susceptibility was determined by the zone of inhibition consistent with the sensitivity chart provided by the manufacturer (HiMedia) and based on CLSI guidelines.^25^

### Study population and period

The study included all patients admitted to the TUTH ICU whose sputum sample was sent for culture and drug susceptibility testing (CDST) from 14 April 2018 to 13 April 2019 and with isolated PABS species. Patients who were admitted for more than 2 calendar days in the health facility before collection of samples for CDST were considered to have a ‘hospital-acquired’ infection, while the rest considered were categorised as ‘community-acquired’.

### Data variables, sources of data and data collection

Data variables included culture growth, presence of Gram-negative bacterial growth, presence of PABS and the antimicrobial resistance patterns of all the sputum samples sent for CDST from the ICU. Age, sex, number of antibiotics used and hospital exit outcomes of those patients with PABS sputum culture were also recorded. Sources of data were microbiology laboratory record files, medical records of ICU patients and the electronic database of ICU patients. A structured data collection proforma including all the study variables was used to extract data.

### Data analysis and statistics

Data were entered using EpiData Entry software v3.1 (Epidata Association, Odense, Denmark). Data were then analysed using EpiData analysis software v2.2.2.186 (EpiData Association) and Stata v11.0 (StataCorp LP, College Station, TX, USA). Numbers and percentages were used to summarise the results of sputum CDST, demographic and clinical characteristics and hospital exit outcomes of adult ICU patients with PABS detected on sputum culture. Fisher’s exact test and χ^2^ test were used to assess the association between demographic and clinical characteristics with resistance to at least one antibiotic. Levels of significance was set at 5% (*P* < 0.05).

### Ethics approval

Ethics approval was obtained from Institutional Review Committee (IRC) of the Institute of Medicine (IOM) of TUTH, Kathmandu, Nepal, and the Union Ethics Advisory Group of the Centre for Operational Research at the International Union Against Tuberculosis and Lung Disease, Paris, France (EAG 06/20). The ethics committee waived the need for informed consent because data were collected from hospital records. The database did not include personal identifiers.

## RESULTS

**FIGURE 1:**
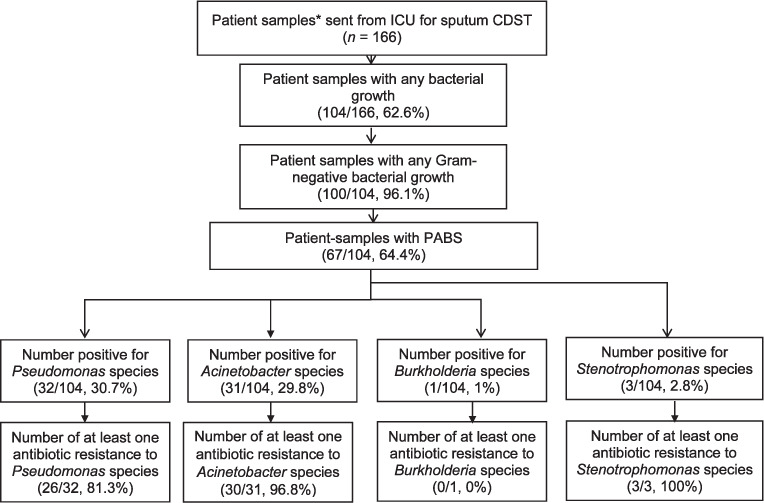
The number and proportion with bacterial growth, Gram-negative bacteria and PABS among adult patients admitted to the ICU and undergoing blood culture at a tertiary care hospital in Kathmandu from April 2018 to April 2019. *Each patient contributes multiple sputum samples. PABS = Pseudomonas, Acinetobacter, Burkholderia and Stenotrophomonas; CDST = culture and drug susceptibility testing.

Of the 166 sputum culture samples from patients admitted to the ICU during study reference period, 63% (104/166) were positive for bacterial growth (Figure 1). Of those sputum cultures with bacterial growth, 64% (67/104) were positive for PABS. Among all the positive cultures, 31% (32/104) were due to *Pseudomonas* and 30% (31/104) due to *Acinetobacter*, while only 3% (3/104) were caused by *Stenotrophomonas*.

The demographic and clinical characteristics of those patients with PABS-related LRTIs are shown in Table 1. Of the total, 97% of patients required ventilator support and central venous line management. The minimum ICU stay was 2 days, with a maximum of 43 days. The proportion of infections acquired in the hospital was higher than that from the community (58% and 42%, respectively). In addition, because of the lack of consistent documentation, it was not possible to accurately determine the number of antibiotics patients had been on prior to obtaining sputum samples.

**TABLE 1:** Demographic and clinical characteristics associated with resistance to at least one antibiotic against PABS among adult patients admitted to the ICU and detected with PABS on sputum culture at a tertiary care hospital in Kathmandu, Nepal, 14 April 2018–15 April 2019

Characteristics	Total		Resistance to at least one antibiotic		*P* value
	
	*N*	(%)^*^	*n*	(%)^†^	
Total					
Age, years (*n* = 63)^‡^	67	(100.0)	59	(88.1)	
18–29	9	(14.3)	8	(88.9)	0.812^§^
30–44	12	(19.0)	11	(91.7)	
45–59	22	(34.9)	18	(81.8)	
⩾60	20	(31.7)	18	(90.0)	
Sex					
Male	47	(70.1)	41	(87.2)	
Female	20	(29.9)	18	(90.0)	0.749§
Reason for admission					
Medical	42	(62.7)	37	(88.1)	
Surgical	25	(37.3)	22	(88.0)	0.991§
Origin of infection^¶^					
Hospital-acquired	39	(58.2)	35	(89.7)	
Community-acquired	28	(41.8)	24	(85.7)	0.616§
Ventilator support^#^					
Yes	59	(96.7)	51	(86.4)	
No	2	(3.3)	2	(100.0)	1.000^**^
Central line cannulation^#^					
Yes	59	(96.7)	51	(86.4)	
No	2	(3.3)	2	(100.0)	1.000^**^
Number of antibiotics used^#^					
Uncertain	57	(85.1	51	(89.5)	
1	2	(3.0)	2	(100.0)	0.574§
2	5	(7.5)	4	(80.0)	
⩾3	3	(4.5)	2	(66.7)	

^*^ Column percentage.

^†^ Row percentage.

^‡^ Information on age was available for only 63 patients.

^§^ χ^2^ test.

**¶** Those who were admitted for more than 2 calendar days in health facility before collection of samples for culture and drug susceptibility test were considered as ‘Hospital origin’ and the rest considered as ‘Community origin’.

**#** Prior to collection of sputum for CDST.

^**^ Fisher’s Exact test.

PABS = *Pseudomonas*, *Acinetobacter*, *Burkholderia* and *Stenotrophomonas*; ICU = intensive care unit; CDST = culture and drug susceptibility testing.

The frequency of antibiotic resistance to high-use antibiotics for *Pseudomonas* was found to be as follows: levofloxacin, 61%, amikacin, 50% and cefepime, 50% (Table 2). For patients with *Acinetobacter*, antibiotic resistance was found to be as follows: cefepime, 95%, imipenem, 92% and levofloxacin, 86%. Of those with *Stenotrophomonas*, 50% were resistant to cefoperazone sulbactam, and 33% to both levofloxacin and tigecycline. There was only one sputum culture-positive sample for *Burkholderia* for which CDST results was not recorded.

**TABLE 2:** The drug resistance pattern of PABS among adult patients admitted to the ICU and undergoing sputum culture at a tertiary care hospital in Kathmandu, Nepal, 14 April 2018–15 April 2019*

Drugs	*Pseudomonas*			*Acinetobacter*			*Stenotrophomonas*		
		
	Test	*n*	(%)^†^	Test	*n*	(%)^†^	Test	*n*	(%)^†^
Total	32			31			3		
Amikacin	28	14	(50.0)	29	22	(75.9)	3	3	(100)
Ampicillin-sulbactam	—	—	—	17	8	(47.1)	2	2	(100.0)
Cefoperazone sulbactam	16	5	(31.2)	19	15	(78.9)	2	1	(50.0)
Cefepime	12	6	(50.0)	19	18	(94.7)	2	0	(0.0)
Ceftazidime	24	13	(52.2)	26	22	(84.6)	2	0	(0.0)
Colistin sulphate	23	4	(17.4)	29	3	(10.3)	2	0	(0.0)
Ciprofloxacin	28	16	(57.1)	28	23	(82.1)	2	0	(0.0)
Cotrimoxazole	16	16	(100.0)	22	20	(90.9)	3	0	(0.0)
Gentamycin	26	11	(42.3)	27	24	(88.9)	2	2	(100)
Imipenem	23	11	(47.8)	24	22	(91.7)	2	2	(100)
Levofloxacin	28	17	(60.7)	28	24	(85.7)	3	1	(33.3)
Meropenem	21	10	(47.6)	26	22	(84.6)	2	2	(100)
Piperacillin tazobactam	24	9	(37.5)	22	17	(77)	3	3	(100.0)
Polymyxin B	26	2	(7.7)	30	2	(6.7)	3	0	(0.0)
Tigecycline	—	—	—	15	10	(66.7)	3	1	(33.3)

^*^As there was only one case of *Burkholderia* in our study, CDST results for this case was not recorded and it has not been included in this table, although this has been discussed in the Results section.

^†^Column percentage.

PABS = *Pseudomonas*, *Acinetobacter*, *Burkholderia* and *Stenotrophomonas*; ICU = intensive care unit; CDST = culture and drug susceptibility testing.

**FIGURE 2:**
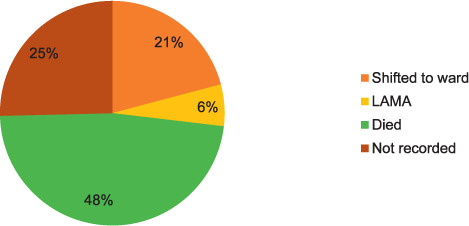
ICU discharge outcomes of the adult patients admitted to the ICU and detected with PABS using sputum culture at a tertiary care hospital in Kathmandu, Nepal, 14 April 2018–15 April 2019. LAMA = leave against medical advice.

The mortality rate in patients with PABS-related LRTI was 48% (32/67) (Figure 2). However, clinical outcomes were not available for 25% of patients, and 6% of patient left against medical advice.

## DISCUSSION

NFGNB-related infections have been emerging in recent years despite increasing awareness and infection prevention control measures among hospitalised patients.^26^,^27^ Identification of these organisms and the underlying antimicrobial resistance could play a key role in rationalising the use of antimicrobial agents, especially within ICU contexts, where broad-spectrum antibiotic use is frequent.

This study reports the magnitude and pattern of antimicrobial resistance of PABS species in sputum samples of patients admitted to the adult ICU of a tertiary care hospital in Kathmandu, Nepal. The key findings were 1) the proportion of sputum cultures with bacterial growth with PABS species was 64%, 2) *Pseudomonas* and *Acinetobacter* were the most commonly isolated PABS species, 3) resistance to at least one antibiotic among *Pseudomonas* and *Acinetobacter* was seen in respectively 81% and 97% of tested samples, and 4) almost half (48%) of the patients with PABS infection died during hospitalisation.

NFGNB have been of less concern, but have emerged as important nosocomial pathogens in recent years.^16^ Our study shows a high prevalence of *Pseudomonas* and *Acinetobacter*, which is consistent with previous studies by Chawla et al. (57% and 39%, respectively)^28^ in India and Yadav et al. (46% and 47%, respectively)^18^ in Nepal.

The study from India also reported isolation of *Burkholderia* (4 samples) and *Stenotrophomonas* (15 samples) among 5,056 samples with bacterial growth, which was lower than the current study (1% and 3%, respectively).^19^ *Burkholderia* was isolated from 5% of the samples in the previous study from Nepal.^18^

A study conducted by Erbay et al. in Turkey showed a lower prevalence of *Pseudomonas* (23%) and *Acinetobacter* (12%) among the patients admitted in ICU,^29^ and mechanical ventilation was found to be a strong risk factor. We found that 97% of patients with PABS-positive cultures were on ventilator support. These results are consistent with the well-known fact that mechanical ventilation is a key risk factor for PABS infection.^18^,^30^ Another study conducted in the same hospital in 2015 by Parajuli et al. reported a lower prevalence of *Pseudomonas* (12%), but higher prevalence of *Acinetobacter* (38%) among patients with hospital-acquired pneumonia.^16^

We found a mortality rate of 48% among patients with PABS-associated LRTI. This rate was likely to be higher, as it was difficult to be more accurate due to incomplete outcomes data. Our findings are consistent with previously reported ventilator-associated pneumonia mortality rates secondary to *Pseudomonas* and *Acinetobacter* infection, which ranged from 27% to 76%.^31^

NFGNB are known to produce extended spectrum β-lactamase and metallo-β-lactamase, which are innately resistant to many antibiotics.^32^ In our study, we have reported high resistance to many commonly used antibiotics. *Acinetobacter* isolates were found to be highly resistant to cefepime (94.7%), imipenem (91.7%), levofloxacin (86%), gentamicin (89%) and amikacin (76%), which was similar to the findings by Parajuli et al.^16^ Moreover, there is emerging resistance to imipenem by *Acinetobacter* as shown by the SENTRY Study conducted in 2011 by Gales et al.^33^ The increased prevalence of this organism could be due to high chance of acquisition of a resistance gene and their ability to persist and multiply in hospital environments. Key to limiting further emergence and spread will require the maintenance of high pharmacovigilance and infection control practices.^34^ In our study, we observed that *Pseudomonas* isolates were also found to be highly resistant to amikacin (50%), levofloxacin (61%) and ceftazidime (52%), which was similar to a previous study from India.^35^

*Stenotrophomonas* species were isolated from only three samples in our study. Of those patients with *Stenotrophomonas*-related infections, there was one mortality. Although not specifically examined in our study, these findings suggest that *Stenotrophomonas* may be evolving into an extensively drug-resistant (XDR) or pan drug-resistant (PDR) pathogen within the ICU at TUTH. This could potentially lead to increased rates of ventilator-associated pneumonia that would be refractory to almost all available therapeutics with high mortality risk. Further study in other similar settings need to be conducted to better understand these findings and provide ongoing surveillance for increasing emergence within Nepal.

The strengths of this study include the following: 1) all sputum samples were from an academic, tertiary ICU submitted for culture during a 12-month period, 2) STROBE guidelines were used to ensure the completeness and quality of reporting.^36^

Study limitations included the relatively small sample size. Patient-specific data from medical records such as previous hospitalisation, underlying comorbidities, previous antimicrobial therapy, number of sputum samples during ICU stay and reason for admission were frequently incomplete, limiting further exploration of possible associated risk factors. As antibiotic susceptibility selection was based on CLSI guidelines, all antibiotics were not tested for susceptibility across all samples. Finally, the data analysis did not allow us to determine the proportion of isolates that were MDR or XDR. These crucial aspects merit further research.

We found that *Pseudomonas* and *Acinetobacter* were the most common NFGNB isolated in patients admitted to the TUTH ICU. In addition, although the prevalence of *Stenotrophomonas* infection currently appears low, the degree of AMR is alarming. Knowledge of resistance patterns to commonly used therapeutic regimens for these organisms can help guide clinical practice for choosing empirical treatment against PABS in the critically ill. This will hopefully help reduce the development of further AMR within the ICU setting. The results of our study need to be validated by others, and broader surveillance measures should be implemented in Nepal and the surrounding SEAR in order to monitor and avert additional emergence of drug resistance. Local, regional and national attention to this growing challenge is needed to mitigate further AMR, failing which patient outcomes will likely worsen with far reaching social and economic impact.

In conclusion, *Pseudomonas* and *Acinetobacter* were the most common NFGNB organisms isolated from sputum specimens among the patients admitted to the ICU of a tertiary hospital in Kathmandu, Nepal. *Stenotrophomonas* isolates that have been reported from other parts of the world were also demonstrated to be present in this ICU and showed high-level of resistance to commonly used broad-spectrum antibiotics. Our findings add to the growing evidence of emerging antibiotic resistance within critical care settings in SEAR, and highlight the need for increased support for surveillance, antibiotic stewardship and education.
